# Prevention of HIV-1 TAT Protein-Induced Peripheral Neuropathy and Mitochondrial Disruption by the Antimuscarinic Pirenzepine

**DOI:** 10.3389/fneur.2021.663373

**Published:** 2021-06-15

**Authors:** May Madi Han, Katie E. Frizzi, Ronald J. Ellis, Nigel A. Calcutt, Jerel Adam Fields

**Affiliations:** ^1^Department of Pathology, University of California, San Diego, La Jolla, CA, United States; ^2^Department of Neuroscience, University of California, San Diego, La Jolla, CA, United States; ^3^Department of Psychiatry, University of California, San Diego, La Jolla, CA, United States

**Keywords:** HIV, tat, neuropathy, mitochondrial dysfunction, sciatic nerve, pirenzepine

## Abstract

HIV-associated distal sensory polyneuropathy (HIV-DSP) affects about one third of people with HIV and is characterized by distal degeneration of axons. The pathogenesis of HIV-DSP is not known and there is currently no FDA-approved treatment. HIV trans-activator of transcription (TAT) is associated with mitochondrial dysfunction and neurotoxicity in the brain and may play a role in the pathogenesis of HIV-DSP. In the present study, we measured indices of peripheral neuropathy in the doxycycline (DOX)-inducible HIV-TAT (iTAT) transgenic mouse and investigated the therapeutic efficacy of a selective muscarinic subtype-1 receptor (M_1_R) antagonist, pirenzepine (PZ). PZ was selected as we have previously shown that it prevents and/or reverses indices of peripheral neuropathy in multiple disease models. DOX alone induced weight loss, tactile allodynia and paw thermal hypoalgesia in normal C57Bl/6J mice. Conduction velocity of large motor fibers, density of small sensory nerve fibers in the cornea and expression of mitochondria-associated proteins in sciatic nerve were unaffected by DOX in normal mice, whereas these parameters were disrupted when DOX was given to iTAT mice to induce TAT expression. Daily injection of PZ (10 mg/kg s.c.) prevented all of the disorders associated with TAT expression. These studies demonstrate that TAT expression disrupts mitochondria and induces indices of sensory and motor peripheral neuropathy and that M_1_R antagonism may be a viable treatment for HIV-DSP. However, some indices of neuropathy in the DOX-inducible TAT transgenic mouse model can be ascribed to DOX treatment rather than TAT expression and data obtained from animal models in which gene expression is modified by DOX should be accompanied by appropriate controls and treated with due caution.

## Introduction

HIV has infected over 37 million people worldwide. The introduction of combined antiretroviral therapy (cART) has been successful in suppressing viral load, prolonging lifespan and improving quality of life of HIV-infected individuals. However, extending life span with cART has been accompanied by an increased prevalence of HIV-associated distal sensory polyneuropathy (HIV-DSP), which now affects over one third of patients with HIV (1). HIV-DSP is initially perceived in the feet and may progress proximally to the legs and hands, with patients experiencing a range of symptoms including loss of sensation to touch, heat or vibration, numbness, tingling and burning pain and allodynia (2). HIV-DSP is characterized by distal degeneration of sensory axons in a dying-back pattern (1, 3) and there is loss of small unmyelinated intraepidermal nerve fibers (IENF) in skin biopsies from distal legs of patients with HIV-DSP (4). The pathogenesis of HIV-DSP has been associated with neurotoxicity of both HIV-associated proteins (3, 5, 6) and cART (7–10). There is no FDA-approved treatment for HIV-DSP and patients exhibiting pain are frequently treated with agents used for other neuropathic pain conditions such as gabapentin, lidocaine gel, capsaicin cream and opioids (2, 11).

One of the HIV viral proteins implicated in HIV-DSP is HIV trans-activator of transcription (TAT). This protein is essential for efficient HIV viral replication and is secreted by HIV-infected microglia, macrophages and astrocytes (6, 12–14). TAT is detectable in the cerebrospinal fluid and peripheral blood samples of HIV-patients (15, 16). The pathogenic role of TAT has been investigated using doxycycline-inducible TAT (iTAT) transgenic mouse models (6). The two predominant models in the field differ only by the number of TAT gene insertions into the genome, with mice having either multiple (6) or single copies (17). With doxycycline (DOX) treatment, the iTAT-tg mouse models express TAT under the glial fibrillary acidic protein (GFAP) promoter in astrocytes at a concentration comparable to that detected in HIV patients on cART (6, 18). These iTAT mice develop cognitive deficits and CNS neuropathologies similar to those seen in HIV patients such as astrogliosis, loss of dendritic spines, neuronal apoptosis and increased infiltration of activated monocytes and T-lymphocytes (6, 19, 20). The pathogenic mechanism of TAT-induced neuropathology has been linked to disruption of mitochondria (14), including increased fission (21, 22).

RNA transcripts for Tat have been detected in the peripheral nervous system of the iTAT tg models, probably transcribed by the GFAP-expressing satellite and Schwann cells (17, 23). However, despite extensive studies on how TAT affects the CNS, few studies have examined the consequence of TAT in the PNS. It has been argued that mitochondrial energy deficits in distal terminals of sensory neurons lead to degeneration or retraction of these regions (24). Mitochondrial dysfunction has been implicated in the pathogenesis of neuropathic pain including mechanical allodynia seen in HIV patients (25, 26) and contributes to a variety of peripheral neuropathies (25), including those associated with diabetes (27–29), chemotherapy (30) and HIV (31). Thus, ameliorating mitochondrial dysfunction may offer an approach to treating both the degenerative and painful aspects of HIV-DSP.

We have previously shown that adult sensory neurons are under metabolic constraint mediated by activation of the muscarinic subtype-1 receptor (M_1_R) (32). Pharmacologically inhibiting M_1_R with the selective antagonist pirenzepine (PZ) activates the AMPK/PGC-1α pathway, enhances mitochondrial function and promotes neurite outgrowth *in vitro*. PZ treatment also prevented and/or reversed multiple indices of diabetic and chemotherapy-induced peripheral neuropathies in rodent models of these conditions (7, 33, 34). Most pertinent to our present studies, topical administration of the specific M_1_R antagonist MT7 to the eye both prevented and reversed corneal sensory nerve loss caused by topical delivery of the neurotoxic HIV envelope protein gp120 (32). We have therefore extended investigation of the therapeutic potential of M_1_R antagonism against HIV-DSP by determining efficacy of systemic PZ against functional and structural disorders of peripheral nerve caused by TAT expression using the iTAT-tg mouse model.

## Methods

### Animals

All animal procedures were approved by the Institutional Animal Care and Use Committee at the University of California San Diego. Animals were housed in an AALAC-accredited vivarium in groups of 3–5 per cage on TEK-Fresh bedding (7099, Envigo) under a 12-hour light:dark cycle with free access to food (5001 diet, Purina, USA) and water. Studies were performed in adult male and female 7–8-month-old mice. The iTAT transgenic mouse model requires treatment with the antibiotic DOX to induce TAT protein expression. However, DOX is known to have anti-inflammatory effects (35) and could potentially serve as an independent variable. Thus, two studies were performed—one to assess potential neurotoxic effects of DOX in normal mice and the second to measure neuropathy in DOX-induced TAT expressing mice and the impact of PZ therapy. The first study consisted of male and female C57BL/6J mice treated with vehicle (*n* = 9) or DOX (*n* = 8). DOX was administered daily at 80 mg/kg i.p. for 2 weeks, as behavioral deficits arising from transgene expression in TAT-expressing mice occur within 7 days of DOX treatment (6). We previously showed that 2 weeks of DOX treatment of the iTAT tg mice induces strong expression of the TAT gene from the GFAP promoter, which is expressed in the peripheral nervous system by Schwann cells (36).

The second study used DOX-inducible TAT transgenic mice treated with vehicle (5 males and 3 females, abbreviated as iTAT), DOX (4 males and 3 females, abbreviated as iTAT+DOX) or DOX and PZ (3 males and 3 females, abbreviated as iTAT+DOX+PZ). The iTAT transgenic mice were produced by crossbreeding Teton-GFAP mice and TRE-Tat86 mice (6) and the DOX-induced expression of TAT in the nervous system of this model has been validated (36). TAT was induced by i.p. DOX administration at 80 mg/kg once daily, five times per week for 2 weeks. For the iTAT+DOX+PZ group, PZ was administered at 10 mg/kg *via* subcutaneous injection once daily five times per week. PZ treatment was initiated on the same day as DOX to assess potential neuroprotective effects. For vehicle treatment, 0.9% saline was administered via subcutaneous and intraperitoneal injection.

After 2 weeks of treatment, rotarod performance, electrophysiological function and hind-paw withdrawal threshold to von Frey filaments and heat were measured (week 3 of the study), followed by corneal confocal microscopy (CCM) to quantify corneal sensory innervation (week 4 of the study). Upon completion of CCM, the mice were euthanized and hind-paw foot skin was collected for assessment of intraepidermal nerve fiber density and sciatic nerves for immunoblot analyses.

### Behavioral Tests

#### Response to Heat Stimulation

The function of small sensory fibers in hind-paw skin was measured by recording latency of hind-paw withdrawal to a heat stimulus using a thermal nociception test device (UARD) as described in technical detail elsewhere (37). The temperature of the device glass surface was stabilized at 30°C and the heating rate was 1°C/sec, with a 20 s cut-off. Measurements were made on both hind paws and repeated four times. The median of the four measurements for each hind paw was calculated and thermal response latency of the mouse calculated by averaging the median measurements of left and right hind paws.

#### Response to Von Frey Filaments

The function of large myelinated sensory fibers in hind-paw skin was measured by recording sensitivity to light touch using manual von Frey filaments (range of 0.16 to 6.0 grams of force, Kom Kare, Inc.) as described in detail elsewhere (37). The testing filament sequence was used to calculate the 50% paw withdrawal threshold (in grams of force) for each hind paw exactly as described elsewhere (38).

#### Rotarod

Impaired motor function can disrupt behavioral responses upon sensory stimuli. To ensure that behavioral responses were not due to impaired motor function, motor coordination of the animals was measured using a 1.25 inch diameter rotarod (Stoelting Co.) as described previously (37) with the rate of rotation increased from the starting speed of four rotations per minute (RPM) to a maximum of 40 RPM within 120 s. After 120 s, rods consistently rotated at 40 RPM for another 180 s. One acclimation run was performed before the test run.

### Electrophysiology

To evaluate the function of large myelinated motor nerve fibers, motor nerve conduction velocity (MNCV) was measured as described previously (37). Each mouse was anesthetized with 4% isoflurane in oxygen and the nerve and body temperatures stabilized at 37°C. The grounding platinum-tipped sub-dermal needle electrode (Grass Technologies) was inserted into skin at the back of the neck. Two recording electrodes were inserted into the interosseous muscle between the second and third, and third and fourth toes. The sciatic nerve was stimulated with a PowerLab stimulator set to deliver a 200-mV, 50-μs-duration square-wave stimulus every 2 s. The stimulating electrode was inserted into the ankle at the Achilles tendon and the sciatic notch to record the resulting electromyogram (EMG) containing the M wave at the Achilles tendon (M_achilles_ wave) and at the notch (M_notch_ wave). This process was repeated three times. MNCV was obtained by calculating the difference between M_achilles_ and M_notch_ for three repeats and dividing the median by the distance between the Achilles tendon and the sciatic notch.

### Nerve Structure

#### Intraepidermal Nerve Fiber Density

Analysis of small sensory fibers in the epidermis (intraepidermal nerve fibers, IENF) was performed as described in detail previously (37). Briefly, hind-paw skin was fixed in 4% buffered paraformaldehyde overnight at 4°C then stored in 0.1M sodium phosphate buffer at 4°C before embedding in paraffin blocks. 6 μm sections were cut using a rotary microtome (Leitz, model 1512) and mounted onto glass slides. Mounted tissues were immunostained with antibody against protein gene product (PGP) 9.5 (1:1,000; cat. #7863-0504, AbD Serotec). Quantification of IENF was done using bright field light microscopy at 40 × magnification. All nerve fragments in the epidermis were counted for detection of early IENF terminal loss prior to retraction as far as the dermis (39). The length of the paw skin was traced under a light microscope using Scion Image software and a tracing pad. IENF density was reported as IENF profiles/mm.

#### Corneal Nerves

To visualize sensory innervation of the cornea, each mouse was anesthetized with 4% isoflurane in oxygen before transfer to the platform of a corneal confocal microscope (Heidelberg Retina Tomograph 3 with Rostock Cornea Module). Eye gel (GenTeal^TM^, Novartis) was applied to both eyes to prevent drying and to connect the eyes to the microscope lens. The microscope objective was positioned close to the center of the apex of cornea and the depth adjusted to the beginning of the sub-basal nerve plexus. Using the Rostock Imaging Software, 40 sequential images, with 2 μm spacing, were collected encompassing the corneal epithelium, sub-basal nerve plexus and stroma. After identifying the sub-basal nerve plexus:stromal junction, five consecutive images of the sub-basal nerve layer moving outwards toward the epidermis and the first 10 images of the stromal layer were quantified. Corneal nerves in each image were traced using a digitizing tablet connected to a computer running Image J software (Image Processing Analysis in Java, National Institutes of Health). Corneal nerve density in each image was reported as number of pixels per area.

### ImmunoBlot

Sciatic nerves from all groups in both studies were homogenized in a buffer containing protease inhibitor cocktails (Calbiochem, cat. no. 524624 and 539131) plus 1.0 mmol/L HEPES (Gibco, cat. no. 15630-080), 5.0 mmol/L benzamidine, 2.0 mmol/L 2-mercaptoethanol (Gibco, cat. no. 21985), 3.0 mmol/L EDTA (Omni pur, cat. no. 4005), 0.5 mmol/L magnesium sulfate, 0.05% sodium azide; final pH 8.8 as previously described (7). Samples were pre-cleared by centrifugation at 5,000 × g for 5 min at room temperature. Supernatants were retained as whole lysate and stored at −80°F until use.

After determination of protein content by bicinchoninic acid assay (Thermo Fisher Scientific, cat. no. 23225), lysates were loaded (20 μg total protein/lane) on 4–12% Bis-Tris gels (Invitrogen, cat. no. WG1402BX10) and electrophoresed in 5% HEPES running buffer and transferred onto PVDF membrane with iBlot transfer stacks (Invitrogen, cat. no. IB24001) using NuPage transfer buffer (ThermoFisher Scientific, cat. no NP0006). The membranes were blocked in 5% BSA in phosphate-buffered saline-tween 20 (PBST) for 1 h. Membranes were incubated overnight at 4°C with primary antibodies (TFAM, ThermoFisher Scientific, cat# PA5-23776; Total OxPhos Complex Kit, ThermoFisher Scientific, cat# 458099; DNM1L Santa Cruz Biotechnology; sc-32989). Following visualization, blots were stripped and probed with a mouse monoclonal antibody against β-actin (ACTB; Sigma Aldrich, cat. no. A5441) in blocking buffer as a loading control. All blots were washed in PBST then incubated with species-specific IgG conjugated to HRP (American Qualex, cat. no. A102P5) diluted 1:5,000 in PBST and visualized with SuperSignal West Femto Maximum Sensitivity Substrate (ThermoFisher Scientific, cat. no. 34096). Images of protein bands were analyzed by semi-quantitative analysis using the VersaDoc gel imaging system and Quantity One software (Bio-Rad). The densitometry of TFAM, OXPhos, and DNM1L bands were normalized to densitometry of ACTB.

### Statistical Analysis

All studies, assays and measurements were performed on coded animals and tissues by observers unaware of the codes. Within group comparisons over time were made by one-way ANOVA or two-way ANOVA with repeat measures followed by Dunnett's *post hoc* test. Between group comparisons were made by unpaired *t* test or one-way ANOVA with Tukey's or Dunnett's *post hoc* test, as indicated.

## Results

### Body Weight and Sensorimotor Function

**Figure 1:**
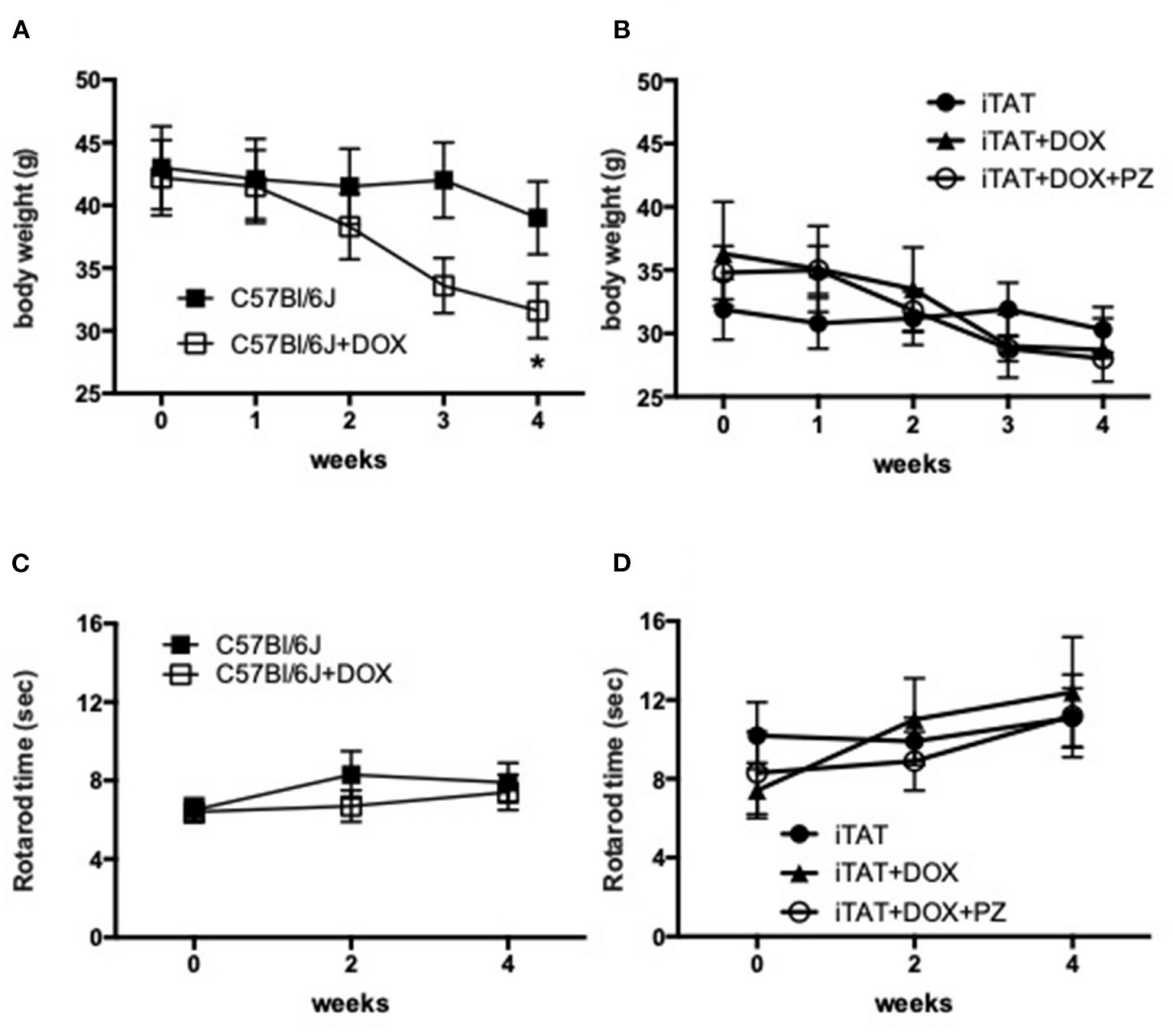
Body weight and sensorimotor function. **(A)** Body weight in C57Bl6/J mice treated with vehicle (filled squares) or DOX (open squares). **(B)** Body weight in TAT transgenic mice treated with vehicle (filled circles), DOX (filled triangles) or DOX and PZ (open circles). **(C)** Rotarod performance in C57Bl6/J mice treated with vehicle (filled squares) or DOX (open squares). **(D)** Rotarod performance in TAT transgenic mice treated with vehicle (filled circles), DOX (filled triangles) or DOX and PZ (open circles). Data are group mean of *N* = 6–9/group ± SEM. Statistical comparisons by two way repeat measures ANOVA followed by Dunnett's *post-hoc* test. *p < 0.05 vs. within group time 0.

Body weight and rotarod performance were assessed before and after DOX treatment to determine any potential systemic effects of DOX. In normal C57Bl/6J mice, DOX treatment decreased body weight over time so that by study end mice were significantly (*p* < 0.05) lighter than before onset of treatment (onset = 42.2±8.6 vs. final = 31.6 ± 6.2 g; mean ± SD; Figure 1A). Vehicle treatment did not change body weight in iTAT mice over time (onset = 31.9 ± 6.7 vs. final =30.3 ± 5.1 g; mean ± SD; Figure 1B), whereas DOX treatment caused a trend to loss of body weight over time (onset = 36.3 ± 10.9 vs. final = 28.7 ± 6.5 g; mean ± SD; Figure 1B) that was not prevented by PZ treatment (onset = 34.8 ± 5.2 vs. final = 28.0 ± 1.6; mean ± SD; Figure 1B). Sensorimotor coordination in normal C57Bl/6J mice, as assessed by the rotarod test, did not change following daily DOX treatment for 2 weeks (Figure 1C). Similarly, there was no change in rotarod performance over time in iTAT mice, whether treated with vehicle, DOX or DOX+PZ (Figure 1D). Therefore, despite a DOX-induced loss of weight, sensorimotor function of DOX-treated mice remained intact, allowing valid measures of behavioral responses in subsequent sensory assays.

### Stimulus-Evoked Behavior and Nerve Electrophysiology

**Figure 2:**
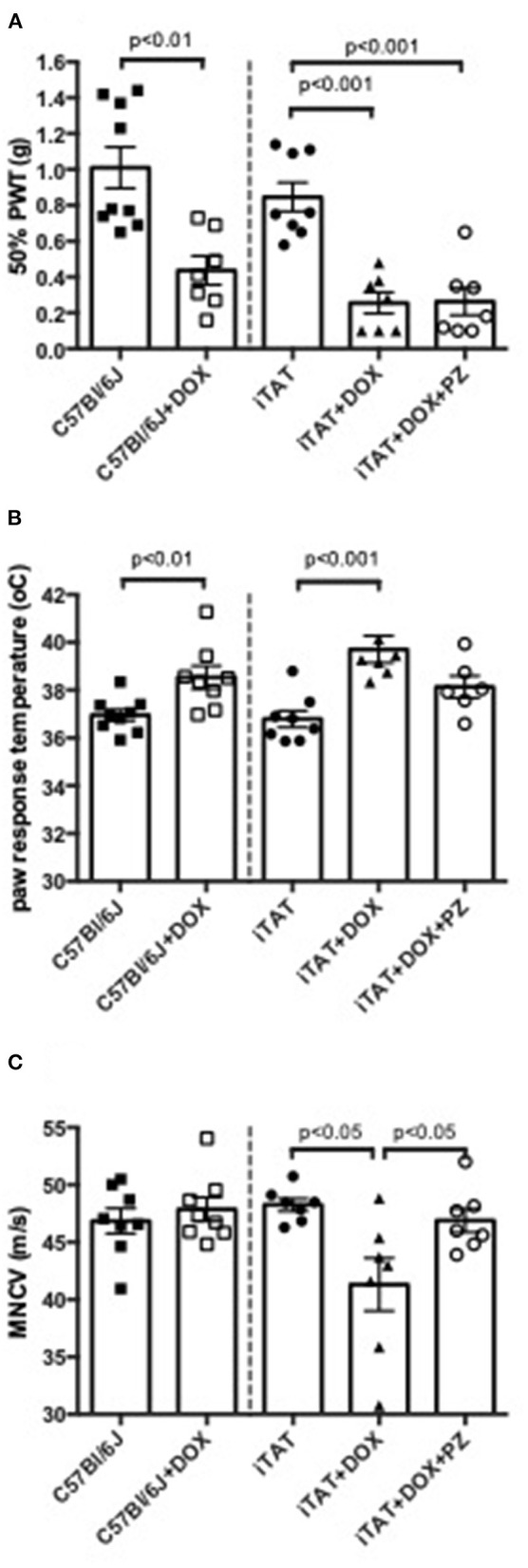
Stimulus-evoked behavior and nerve electrophysiology. **(A)** Paw response to von Frey filaments, **(B)** temperature of paw withdrawal to escalating heat and **(C)** motor nerve conduction velocity in C57Bl6/J mice treated with vehicle or DOX and iTAT transgenic mice treated with vehicle, DOX or DOX and PZ Data are group mean of *N* = 6–9/group ± SEM. Statistical comparisons by unpaired *t* test (left sub-panel) or one-way ANOVA followed by Tukey's *post-hoc* test (right sub-panel).

In normal C57Bl/6J mice, DOX treatment significantly (*p* < 0.01) decreased 50% paw response threshold (C57 = 1.01 ± 0.34 vs. C57 + DOX = 0.44 ± 0.21g; mean ± SD; Figure 2A, left sub-panel) and significantly (*p* < 0.01) increased paw response to heat (C57 = 36.9±0.7 vs. C57+DOX = 38.5±1.3°C; mean ± SD; Figure 2B, left sub-panel) whereas MNCV was unaffected by DOX treatment (C57 = 46.9 ± 3.1 vs. C57 + DOX = 47.9 ± 2.9 m/s; mean ± SD; Figure 2C, left sub-panel). DOX treatment of iTAT mice also induced significant (*p* < 0.001) tactile allodynia (iTAT = 0.84 ± 0.23 vs. iTAT+DOX = 0.25 ± 0.16 g; mean ± SD) and heat hypoalgesia (iTAT = 37.2 ± 1.7 vs. 39.2±1.9°C; mean ± SD) when compared to vehicle treated iTAT mice (Figures 2A,B, right sub-panels). This data showed that the DOX treatment regime used impacted sensory nerve function, thereby precluding determination as to whether tactile allodynia and heat hypoalgesia observed in DOX treated iTAT mice were due to TAT expression or DOX. In contrast to C57 mice, iTAT mice treated with DOX developed significant (*p* < 0.05) MNCV slowing when compared to vehicle-treated iTAT mice (iTAT = 47.1 ± 3.5 vs. iTAT + DOX = 41.3 ± 6.1 m/s; mean ± SD; Figure 2C, right sub-panel), suggesting that MNCV slowing was likely due to TAT expression and not the DOX regime *per se*. Concurrent treatment of iTAT mice with DOX and PZ did not alter tactile allodynia (0.29 ± 0.21 g), whereas it returned paw heat response (37.9 ± 1.3; mean ± SD) and MNCV (46.9 ± 2.7 m/s; mean ± SD) to values that were not significantly different from vehicle-treated iTAT mice (Figures 2A–C, right sub-panel).

### Sensory Nerve Structure

**Figure 3:**
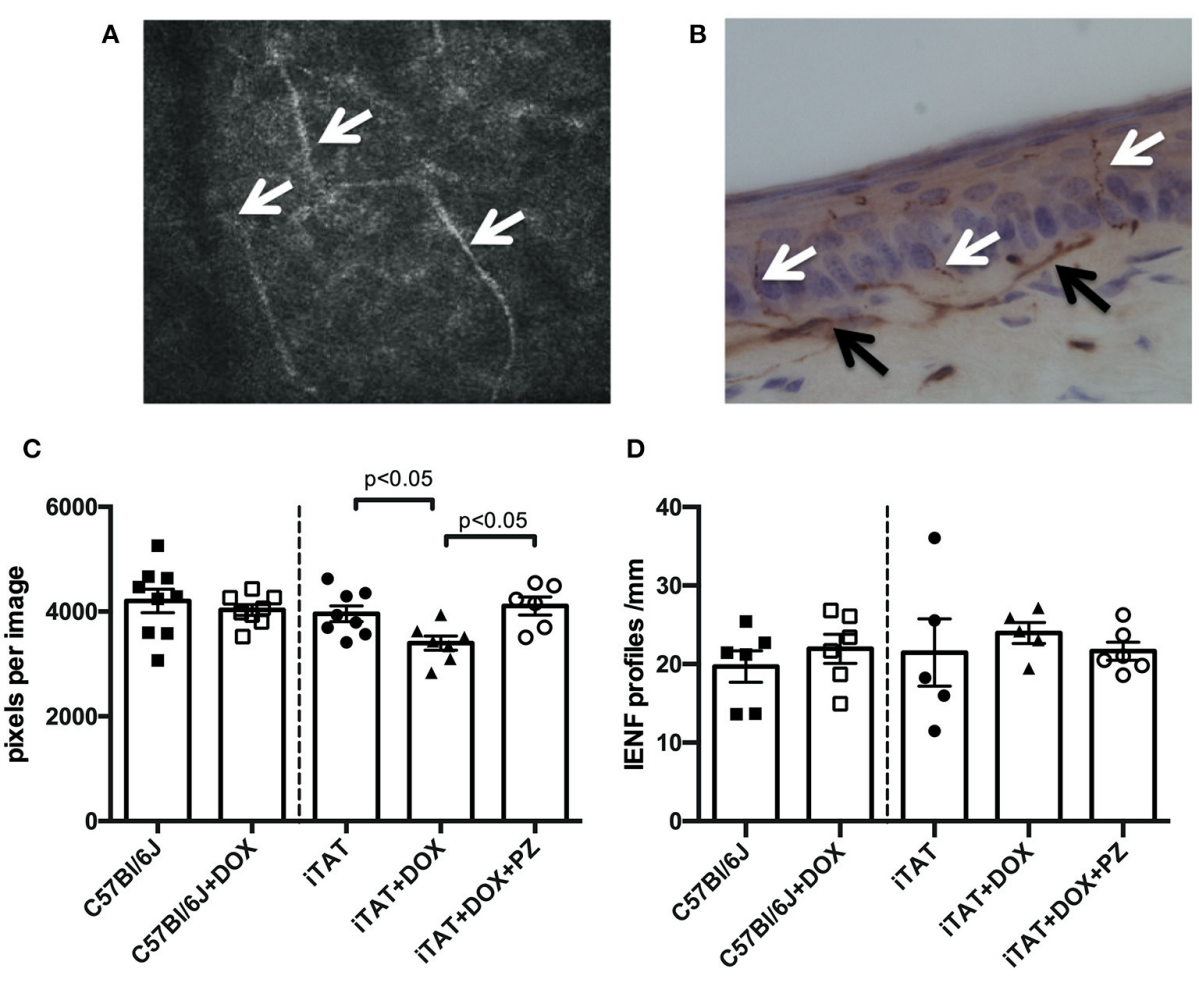
Sensory nerve structure. **(A)** Representative image of mouse corneal sub-basal nerves (white arrowheads). **(B)** Representative image of mouse paw skin dermal (black arrowheads) and intra-epidermal (white arrowheads) PGP9.5+ve nerves. **(C)** Corneal nerve density in C57Bl6/J mice treated with vehicle or DOX and in TAT transgenic mice treated with vehicle, DOX or DOX and PZ. **(D)** Intra-epidermal nerve fiber density of C57Bl6/J mice treated with vehicle or DOX and of TAT transgenic mice treated with vehicle, DOX or DOX and PZ. Data are group mean of *N* = 5–9/group ± SEM. Statistical comparisons by unpaired *t* test (left sub-panel) or one-way ANOVA followed by Tukey's *post-hoc* test (right sub-panel).

Representative images of corneal sub-basal nerves and the dermal and intra-epidermal nerve fibers (IENF) present in hind-paw plantar skin are shown in Figures 3A,B, respectively. In normal C57Bl/6J mice, DOX treatment was without effect on corneal sub-basal nerve density (Figure 3C, left sub-panel) or paw skin IENF density (Figure 3D, left sub-panel) indicating that DOX is not toxic to small fiber structure. DOX-treated iTAT mice had significantly (*p* < 0.05) reduced nerve density in the corneal sub-basal plexus (3,396 ± 359 pixels/image; mean ± SD) compared to vehicle-treated iTAT mice (3,935 ± 425 pixels/image; mean ± SD) and this was prevented by concurrent treatment with PZ (4,106 ± 422 pixels/image; mean ± SD; Figure 3C). Thus, it appears that PZ prevents corneal nerve loss associated with induction of TAT expression. In contrast, neither DOX treatment alone, or in combination with PZ, caused a significant effect on paw skin IENF density of iTAT mice when compared to vehicle-treated iTAT mice (Figure 3D).

### Mitochondrial Proteins in Sciatic Nerve

**Figure 4:**
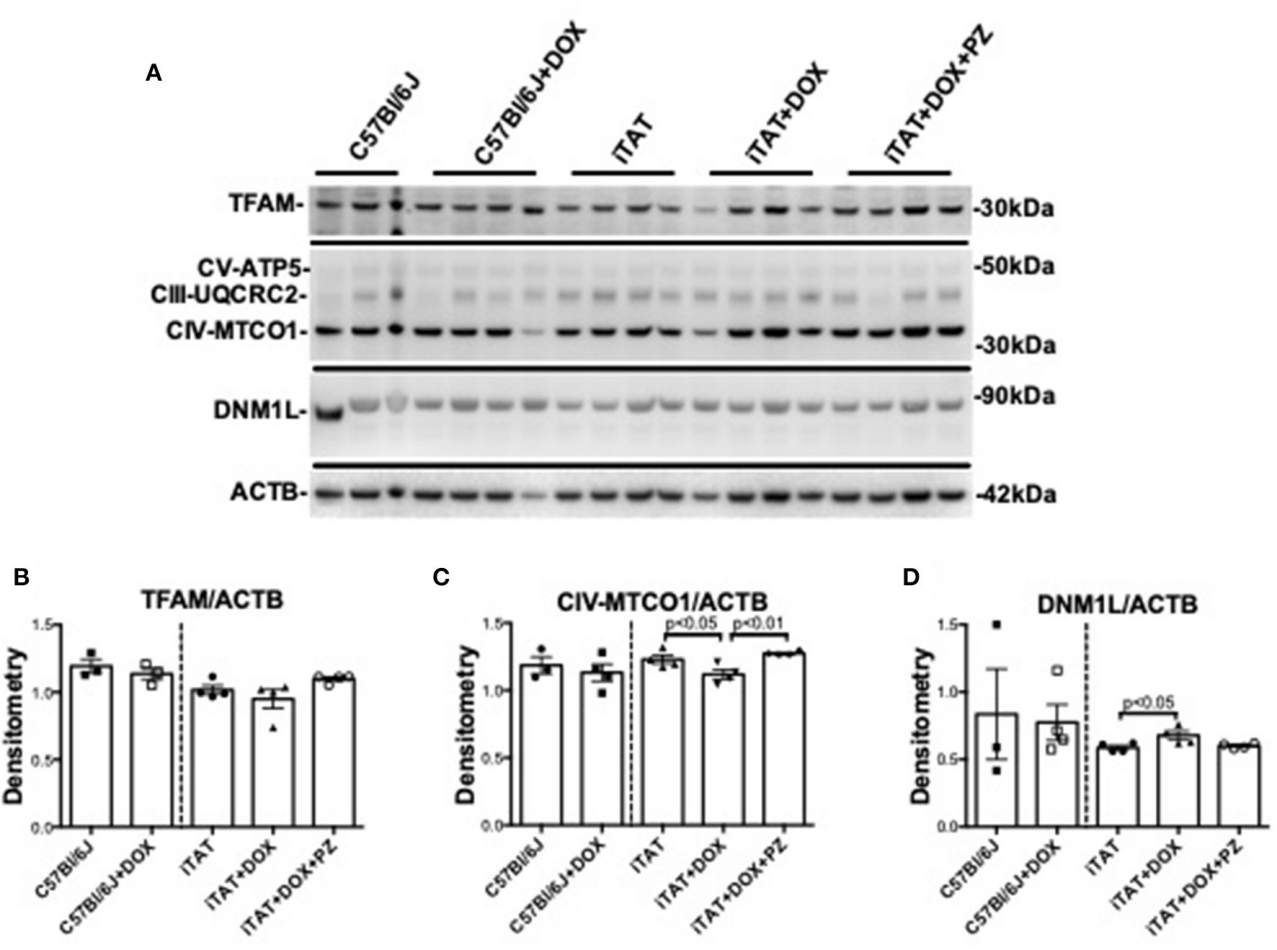
Mitochondrial proteins in mouse sciatic nerve. **(A)** Representative immunoblot for TFAM, proteins of oxidative phosphorylation complexes III, IV, and V, DNM1L and ACTB. **(B)** Densitometric analysis of TFAM normalized to ACTB. **(C)** Densitometric analysis of complex IV(MTC01 sub-unit) protein normalized to ACTB. **(D)** Densitometric analyses of DNM1L protein normalized to ACTB. Data are mean + SEM of *N* = 3 for C57Bl/6J group and *N* = 4/group for remaining groups. Statistical analyses by unpaired test or one-way ANOVA with Tukey's *post-hoc* test.

In light of the DOX-induced physiological changes between C57 and iTAT mice the limited amounts of sciatic were used for exploratory biochemical studies of mitochondrial markers. Sciatic nerve protein lysates were assayed by immunoblot for proteins associated with mitochondrial transcription (TFAM), mitochondrial oxidative phosphorylation at complex III (UQCRC2), complex IV (MTCO1) and complex V (ATP5) and with mitochondrial fission (DNM1L) relative to the housekeeping gene β-actin (ACTB) (Figure 4A) in order to explore the potential role of mitochondrial dysfunction in TAT-induced neuropathy and establish any effects of the PZ intervention on mitochondrial proteins. In C57Bl/6J mice DOX treatment had no effect on any of these mitochondrial-associated proteins (Figures 4A–D, left sub-panels**)** suggesting that DOX alone is not disruptive of mitochondria. DOX treatment was also without impact on TFAM (Figures 4A,B, right sub-panel), CIII-UQCRC2 or CV-ATP5 protein (Figure 4A: quantification not shown). However, in iTAT mice, DOX treatment caused a significant (*p* < 0.05) reduction in CIV-MTCO1 protein (1.11 ± 0.07 AU; mean ± SD) compared to vehicle-treated iTAT mice (1.23 ± 0.06 AU; mean ± SD) and iTAT mice treated with DOX and PZ (1.28 ± 0.02 AU; mean ± SD; Figures 4A,C). DOX treatment of iTAT mice also caused a significant (*p* < 0.05) increase in levels of DNM1L (0.68 ± 0.06 AU; mean ± SD) compared to vehicle-treated iTAT mice (0.58±0.03 AU; mean ± SD) that was also absent in mice treated with PZ (0.60 ± 0.01 AU; mean ± SD; Figures 4A,D). These findings suggested that PZ prevented both the TAT-induced reduced expression of proteins associated with oxidative phosphorylation complex IV and a corresponding in mitochondrial fission.

## Discussion

We investigated the pathogenic role of TAT protein in HIV-DSP using a well-characterized iTAT–tg mouse model and demonstrated that induction of TAT leads to functional and structural indices of motor and sensory neuropathy, accompanied by reduced expression of proteins of the electron transport chain and increased expression of a protein involved in mitochondrial fission. Concurrent treatment with the M_1_R antagonist PZ prevented all indices of mitochondrial dysfunction and peripheral neuropathy. While MNCV, corneal nerves and mitochondrial protein expression co-vary with both TAT expression and PZ therapy, we cannot yet ascribe a causal relationship linking TAT expression, mitochondrial dysfunction and peripheral neuropathy and further mechanistic studies are required to address this possibility.

The trans-activator of transcription (HIV-TAT) protein is an important regulatory protein that allows HIV to replicate efficiently (13). Low levels of TAT protein and viral replication persist in HIV patients on cART despite suppression of the viral load (40, 41). Expression of TAT in the iTAT-tg mouse model has been associated with neuronal dysfunction and damage in the CNS (6, 19, 20) that resembles disorders seen in HIV patients. A recent study conducted with a similar DOX-inducible TAT expressing transgenic mouse model also reported peripheral neuropathy, as indicated by tactile allodynia and reduction of paw IENF density, with no changes in paw thermal withdrawal threshold (17). In our present study we replicated the report of tactile allodynia, but this was accompanied by paw thermal hypoalgesia and normal IENF density. There are a number of differences in the design and analysis of the two studies that could contribute to these discrepancies. Firstly, we provided DOX by daily ip injection for 2 weeks whereas the prior report provided DOX in the diet for 9 weeks. Our more aggressive DOX regimen induced weight loss but not impairment of sensorimotor coordination as measured by rotarod performance, so that sensory assays relying on limb withdrawal responses remain valid. Our study design also allowed us to identify any DOX-associated impact on the PNS independent of TAT induction and demonstrated that both tactile allodynia and paw thermal hypoalgesia in DOX-treated iTAT-tg mice also occurred in DOX-treated non-tg mice and are thus likely attributable to the DOX regimen rather than TAT expression. The prior study limited their comparison to normal mice treated with DOX vs. iTAT-tg mice treated with DOX and thus could not resolve any impact of the DOX regimen *per se*. Further, loss of IENF was detected after 6 weeks of DOX exposure in iTAT-tg mice, but not 8 days (17). Our failure to detect this disorder could reflect the shorter duration of DOX exposure and TAT induction (2 weeks) or withdrawal of DOX for 2 weeks prior to tissue collection that could allow IENF time to regenerate. Finally, there is also a report of sex-dependent effects of TAT on responses to inflammatory or neuropathic insults (42) and while the prior report was limited to female mice, we included both sexes. However, in *post-hoc* exploratory analyses we identified no differences in PNS function or structure between sexes.

Despite the above caveats, large fiber MNCV slowing is likely due to TAT expression, as DOX did not affect this parameter in normal mice. To the best of our knowledge, this is the first study to quantify function of large myelinated motor fibers in the iTAT-tg mouse model, although nerve conduction studies have been used for diagnosing peripheral neuropathy in HIV patients (43–46). Similarly, our novel finding of reduced corneal sensory nerve density is not a consequence of DOX exposure *per se* and is consistent with recent reports of reduced corneal nerve density in patients with HIV neuropathy (47, 48). In a previous study we demonstrated that topical delivery of the HIV envelope protein gp120 to the eye of normal mice also leads to loss of corneal sensory nerves (32), so that two HIV-associated proteins linked with neuropathy independently impact corneal nerves. In both cases, PZ was effective at preventing and/or reversing reduced corneal nerve density, which is consistent with its capacity to provide neuroprotection and promote neurite growth in sensory nerves (32). At present we cannot determine whether the efficacy of PZ was via prevention of corneal nerve degeneration, collateral sprouting from surviving neurons or some combination of both and future intervention treatment studies will be required to address this issue. Our findings also indicate that, at least in the DOX-induced iTAT model, MNCV slowing and corneal nerve depletion precede IENF loss. Electrophysiological testing, a widely used neurological diagnostic technique, and non-invasive corneal confocal microscopy therefore have potential as early biomarkers of large and small fiber peripheral neuropathy in HIV patients.

In neuronal cell cultures, TAT damages neurons by inducing mitochondrial depolarization and increasing mitochondrial fission leading to rapid release of reactive oxygen species and smaller mitochondrial size (14). Mitochondrial fission is controlled by a dynamin like protein 1 (DNM1L), which has been shown to be dysregulated in the brains of people with HIV-associated neurocognitive disorders (21, 22). Similar mitochondrial functional and morphological abnormalities have been observed in postmortem brains of iTAT-tg mice (14). Our current findings in sciatic nerves are consistent with recent studies showing that TAT disrupts oxidative phosphorylation and mitochondrial dynamics in multiple cellular models (49, 50). Reversal of TAT-induced mitochondrial alterations after PZ administration may reflect the capacity of PZ to activate ERK/CREB signaling to promote neurite outgrowth in sensory neurons via regulation of microtubule polymerization and mitochondrial trafficking (51). Interestingly, this signaling pathway is also inhibited by TAT protein leading to subsequent neurite retraction (52). Thus, the therapeutic efficacy of PZ against indices of TAT associated HIV-DSP is consistent with its general mitochondrial enhancing and nerve growth-promoting properties.

Our study is limited to the use of a mouse model that expresses only one HIV-associated protein and the use of DOX to induce TAT expression. Although the iTAT-tg mouse model does not illustrate the complex interaction between HIV and HIV proteins that may impact development of HIV-DSP in humans, it does focus attention on one specific protein and its role in clinical features of HIV-DSP such as nerve conduction slowing and reduction of corneal sensory nerve density (2, 47, 48). Appropriate controls in our study also allowed us to segregate the impact of DOX on sensory nerve function. This study is also limited to descriptive analyses of DSP-related symptoms in the iTAT-tg mouse and how PZ affects these symptoms. While the covariance of mitochondrial associated proteins with neuropathy following TAT expression and PZ treatment offers potential pathogenic and therapeutic mechanisms further studies are needed to better understand the physiological and molecular mechanisms involved and establish any causal links.

In summary, TAT expression in DOX-induced TAT transgenic mice produced indices of large and small fiber neuropathy that replicate those seen in clinical HIV-DSP. These deficits were prevented by treatment with the selective M_1_R antagonist, PZ, as were changes in expression of proteins of mitochondrial respiration and fission. It was notable that our DOX regimen independently impacted sensitivity to touch and heat in normal mice suggesting a need for caution and appropriate controls when using this DOX regime to induce expression of proteins of interest.

## Data Availability Statement

The raw data supporting the conclusions of this article will be made available by the authors, without undue reservation.

## Ethics Statement

The animal study was reviewed and approved by Institutional Animal Care and Use Committee at the University of California San Diego.

## Author Contributions

JF, RE, and NC conceived, designed, coordinated the study, and edited the manuscript. MH performed the experiments, analyzed data and wrote the manuscript. KF performed the experiments and analyzed the data. All authors contributed to the article and approved the submitted version.

## Conflict of Interest

NIH STTR award NS105177 is held by JF and WinSanTor Inc. NC is a scientific founder of WinSanTor Inc. and has an equity interest on the company while KF is an employee of WinSanTor Inc. The terms of this arrangement have been reviewed, approved and managed by the University of California San Diego in accordance with its conflict of interest policies. The remaining authors declare that the research was conducted in the absence of any commercial or financial relationships that could be construed as a potential conflict of interest.

## Abbreviations

HIV: human immunodeficiency virus
tg: transgenic
CNS: central nervous system
PNS: peripheral nervous system
HIV-DSP: HIV-associated distal sensory polyneuropathy
TAT: trans-activator of transcription
PZ: pirenzepine
M_1_R: muscarinic subtype-1 receptor
iTAT: doxycycline-inducible HIV-TAT
cART: combined antiretroviral therapy
IENF: intraepidermal nerve fibers
DOX: doxycycline
GFAP: glial fibrillary acidic protein
CCM: corneal confocal microscopy
RPM: rotations per minute
MNCV: motor nerve conduction velocity
SNCV: sensory nerve conduction velocity
EMG: electromyogram
M_achilles_ wave: M wave at the Achilles tendon
M_notch_ wave: M wave at the sciatic notch
PBST: phosphate-buffered saline-tween 20
TFAM: transcription factor A.
